# Situation aware intelligent reasoning during disaster situation in smart cities

**DOI:** 10.3389/fpsyg.2022.970789

**Published:** 2022-08-08

**Authors:** Kiran Saleem, Salwa Muhammad Akhtar, Makia Nazir, Ahmad S. Almadhor, Yousaf Bin Zikria, Rana Zeeshan Ahmad, Sung Won Kim

**Affiliations:** ^1^Department of Software Engineering, University of Lahore, Lahore, Pakistan; ^2^College of Computer and Information Sciences, Jouf University, Sakakah, Saudi Arabia; ^3^Department of Information and Communication Engineering, Yeungnam University, Gyeongsan, South Korea; ^4^Information Technology Department, University of Sialkot, Sialkot, Pakistan

**Keywords:** situation-awareness, smart cities, edge intelligence, disaster management system, intelligent decision support system, BDI mechanism, multi-agent system

## Abstract

Investigating prior methodologies, it has come to our knowledge that in smart cities, a disaster management system needs an autonomous reasoning mechanism to efficiently enhance the situation awareness of disaster sites and reduce its after-effects. Disasters are unavoidable events that occur at anytime and anywhere. Timely response to hazardous situations can save countless lives. Therefore, this paper introduces a multi-agent system (MAS) with a situation-awareness method utilizing NB-IoT, cyan industrial Internet of things (IIOT), and edge intelligence to have efficient energy, optimistic planning, range flexibility, and handle the situation promptly. We introduce the belief-desire-intention (BDI) reasoning mechanism in a MAS to enhance the ability to have disaster information when an event occurs and perform an intelligent reasoning mechanism to act efficiently in a dynamic environment. Moreover, we illustrate the framework using a case study to determine the working of the proposed system. We develop ontology and a prototype model to demonstrate the scalability of our proposed system.

## 1. Introduction

In smart cities, disasters could be natural or man-made that can occur at any time and anywhere (Javed et al., 2021a,b, 2022). Two methods can solve the problem of disaster, the first one is a conventional disaster system, where humans can make their strategies to design a model, and the other one is an agent-based disaster management system (Mahfooz Ul Haque et al., 2021).

In addition, a multi-agent system (MAS) has been widely used in smart city emergencies resulting from natural or human-made disasters (Akhtar et al., 2022). The MAS has a macro perspective, which is the distribution of knowledge and resource reasoning capabilities (Akhtar et al., 2020). We need the belief, desire, intention (BDI) reasoning mechanism in complex environments to define the agent thinking process. BDI reasoning mechanism provides the most natural way to define the agent (Saleem et al., 2022). We must consider computational power or memory limitations to use the multi-agent approach. We do not want an agent's reasoning to take time and not be fast and efficient enough to make decisions promptly. Furthermore, an agent acts in an environment that is dynamic. For example, the environment may change when an agent is engaged with reasoning about something. In that case, we do not want an agent to plan from the beginning again because an agent might spend all the time planning and re-planning, and the action may never get triggered because of a dynamic environment (Georgeff et al., 1998). When we want the system to be adaptive according to its environment, then we use the BDI reasoning mechanism. Literature has revealed tremendous effort in intelligent decision making systems (Abideen et al., 2021); however, situation awareness and BDI reasoning mechanism have been used in route recognition (Baumgartl and Buettner, 2020), to improve the decision making (Buettner and Baumgartl, 2019) and to develop intentional learning (Ramirez and Fasli, 2017) among agents. Consequently, BDI and situation-awareness lead to a better decision-making system regarding energy efficiency, memory efficiency, and optimal software level (Buettner and Baumgartl, 2019). Situation awareness originated in aviation training and is often used in medical care to provide better patient quality and safety. Situation-awareness is about engaging and being entirely aware of the environment (Stanton et al., 2017).

Situation awareness is not just about collecting information from their environment. A person should be capable of observing, understanding, and projecting future outcomes in the environment (Schermer, 2007). Therefore, the formal definition of situation-awareness depends on three terms 1) perception, 2) comprehension, and 3) projection.

Moreover, the smart environment is supported by internet of things (IoT), and IoT has gradually changed in recent years from IoT to artificial intelligence (AIoT) (Shafiq et al., 2020b; Fayyaz et al., 2022). In addition, UAVs have been combined with IoT to improve the conventional methods of agriculture into smart ones (Kumar et al., 2021b; Majid et al., 2022). Besides that, researchers have done a tremendous amount of work to secure the intelligent decision smart systems in transport domains, industrial IoT (IIOT), industrial healthcare systems, etc., where sensitive information cannot be at risk with the usage of blockchain (Javed et al., 2020a,b, 2021c; Kumar et al., 2021a, 2022a,b). Blockchain based methods are increasing in demand nowadays due to their ability to interact with IoT-based smart environments for providing users with added security and privacy (Deebak et al., 2022b), creating new and improved cybersecurity labeling scheme (CLS) (Wang et al., 2021) and providing a system that increases the packet delivery ratio and mobility speed (Deebak et al., 2022a).

By IoT, researchers have also utilized a wireless sensor network approach to transmit the data from sensors to the servers (Mubashar et al., 2022). However, the problem is the range of wireless communication. To resolve this problem, narrowband IoT comes up. NB-IoT supports the 3GPP, which enables the IoT devices to communicate with a flexible range of deployment. It also supports a tremendous number of devices within a single cell. Using the smart environment concept is not only for monitoring but also to control the situation from worsening based on sensor measurements (Lin et al., 2020). NB-IoT has been utilized in mobile computing, disaster management, clinical health, and remote monitoring to name a few (Chen et al., 2019; Xie et al., 2019; Lin et al., 2020).

With the implementation of an intelligent decision-support mechanism and situation-awareness, the autonomous reasoning-based framework, such as BDI for making intelligent and prudent judgments, is still overlooked. We want the system to be sophisticated enough to systematically and dynamically monitor potentially dangerous circumstances, make judgments without human intervention, and notify rescue personnel as quickly as possible before the situation becomes more problematic. To our knowledge, no systematic framework supporting the autonomous reasoning mechanism with the situation-aware approach has yet been devised. We need this method in our crisis management systems so that the system can intelligently determine dangerous conditions without human intervention, adjust itself to the circumstances, and deduce an appropriate strategy to avoid future harm.

Additionally, the NB-IoT approach is not only helpful for range problems but also beneficial in terms of energy consumption and latency problems on the hardware level (Chen et al., 2019). In addition, edge intelligence plays a vital role in disaster management in a smart environment (Shafiq et al., 2020a). Disaster scenarios highly depend on the response time; therefore, the instant reporting of the abnormal event is crucial for quick actions (Muhammad et al., 2019). Moreover, utilizing the IoT approach for smart systems is becoming a promising method for energy (Mehmood et al., 2019; Ahamad et al., 2021). Based on the points mentioned above, increasing edge intelligence is helpful in data availability, analysis, and promptly processing of sensitive data. Hence, this paper introduces a situation-aware edge communication in a smart cities disaster situation that uses a MAS with a BDI reasoning mechanism on the software level, with an NB-IoT approach on a physical level using the situation-awareness method. Moreover, in the future, the proposed framework will be implemented into a smart environment, where the data will be processed on the network's edge. We incorporate ontology for modeling the proposed system and use the case study briefly to demonstrate the working of our proposed formalism and implement a prototypal model to determine the system's behavior. Despite the IoT concept, likewise, the proposed system is based on IIOT. The proposed system is making life convenient for consumers, increasing their safety regarding disaster management, and utilizing edge intelligence.

The structure of the paper is as follows. Section 2 presents the relevant work to the situation-aware edge communication in smart cities disaster situations. Section 3 presents the extensive description of the framework we propose concerning BDI reasoning with situation awareness and NB-IoT. Section 4 discusses the core algorithm of our proposed system. In Section 5, we present the formal structure of our proposed system by OWL 2 ontology. Section 6 discusses the prototypal implementation of the system. Section 7 gives conclusion.

## 2. Background and motivation

Significant work has been done to efficiently handle smart city disaster situations using several computing domains. In Buford et al.'s (2006) paper, authors introduced the BDI SM agent model. The BDI abstract model is based on beliefs, desires, and intentions. According to the authors, the conventional method of BDI only worked with the single-driven event; that is why, the authors utilized the situation-awareness approach to enhance the capabilities of agents.

Foundation for intelligent physical agents (FIPA) was the standard protocol in which the agents could communicate with each other. The authors used the FIPA platforms for interaction among agents. The core of this paper was based on disaster emergencies. In Feng et al.'s (2009) study, the authors proposed an intelligent decision support system based on context-awareness with a situational model for shared situation modeling. The system consisted of agents, customized decision support, rule-based reasoning, event classification action recommendation, and proactive decision making.

In Luqman et al. and Sharmeen et al.'s studies (Luqman and Griss, 2010; Sharmeen et al.'s, 2014), the authors provided a framework and app which helped the rescue teams to be connected, and if the rescue team member had any query, a member could use the application, and the application presented the solution to the current scenario. In Ramchurn et al.'s study (Ramchurn et al., 2015), the authors provided a framework that dealt with the disaster situation. The authors used human, agent, and unmanned aerial vehicles (UAVs) in this scenario. The authors provided a framework that collected the information using crowdsourcing. After launching the UAV, the agents acquired the data to observe the environment in detail. The agent sent the teams to rescue the victims. However, according to our best knowledge, no system has been developed using the BDI reasoning mechanism with situation-awareness and NB-IoT approach that handles 3 to 4 natural disasters simultaneously with handling energy consumption constraints, range flexibility, and optimal planning a few.

## 3. BDI agents-based situation-aware formalism

The motto and determination of the system are “Help victims who are in a disaster situation using the intelligent means in order to save lives.” The proposed system uses BDI reasoning with a situation-aware technique to observe the surroundings without human involvement and instantly benefit the victims.

**Figure 1:**
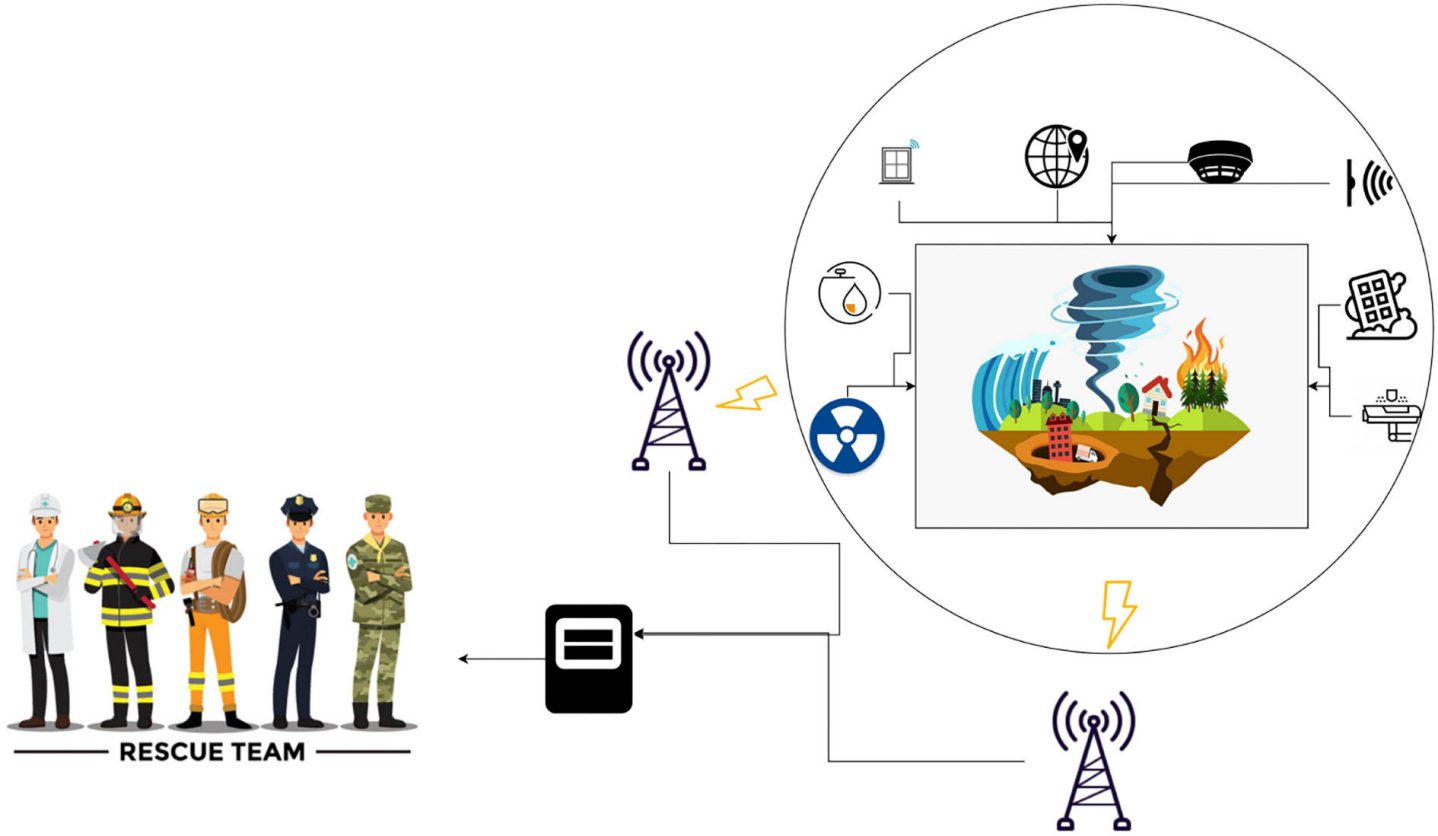
The architecture of the system.

The proposed system consists of an NB-IoT module, WiFi, hardware sensors, and a MAS with situation awareness on a software level. As shown in Figure 1, every sensor transmits the data in a smart environment with an NB-IoT module. If any disaster occurs, the sensors get the data and transfer it to the software level (intelligent decision support system) through service providers, where the multi-agent system acquires the information, performs reasoning on it, and calls the rescue teams.

The crucial role of using an autonomous intelligent agent is to provide the decision support mechanism with situation awareness. Therefore, the system is intelligent enough to make decisions in an effective and timely manner. Due to the dynamic and complex situations, we need an intelligent system to enhance the situation-awareness capability, including a BDI decision support system. BDI has become the most significant part of a multi-agent environment. In the BDI reasoning environment, an agent gets information about the environment. It knows what direction it is heading to achieve the goal because of its belief. The dynamic environment processes information and does not know where the information leads anyone and decides the next course of action is time and energy-consuming. Therefore, we need a goal-directed path where agents cannot process from scratch whenever the situation changes. Hence, the BDI reasoning approach is promising in dynamic situations such as disaster management (Adam and Gaudou, 2016).

**Figure 2:**
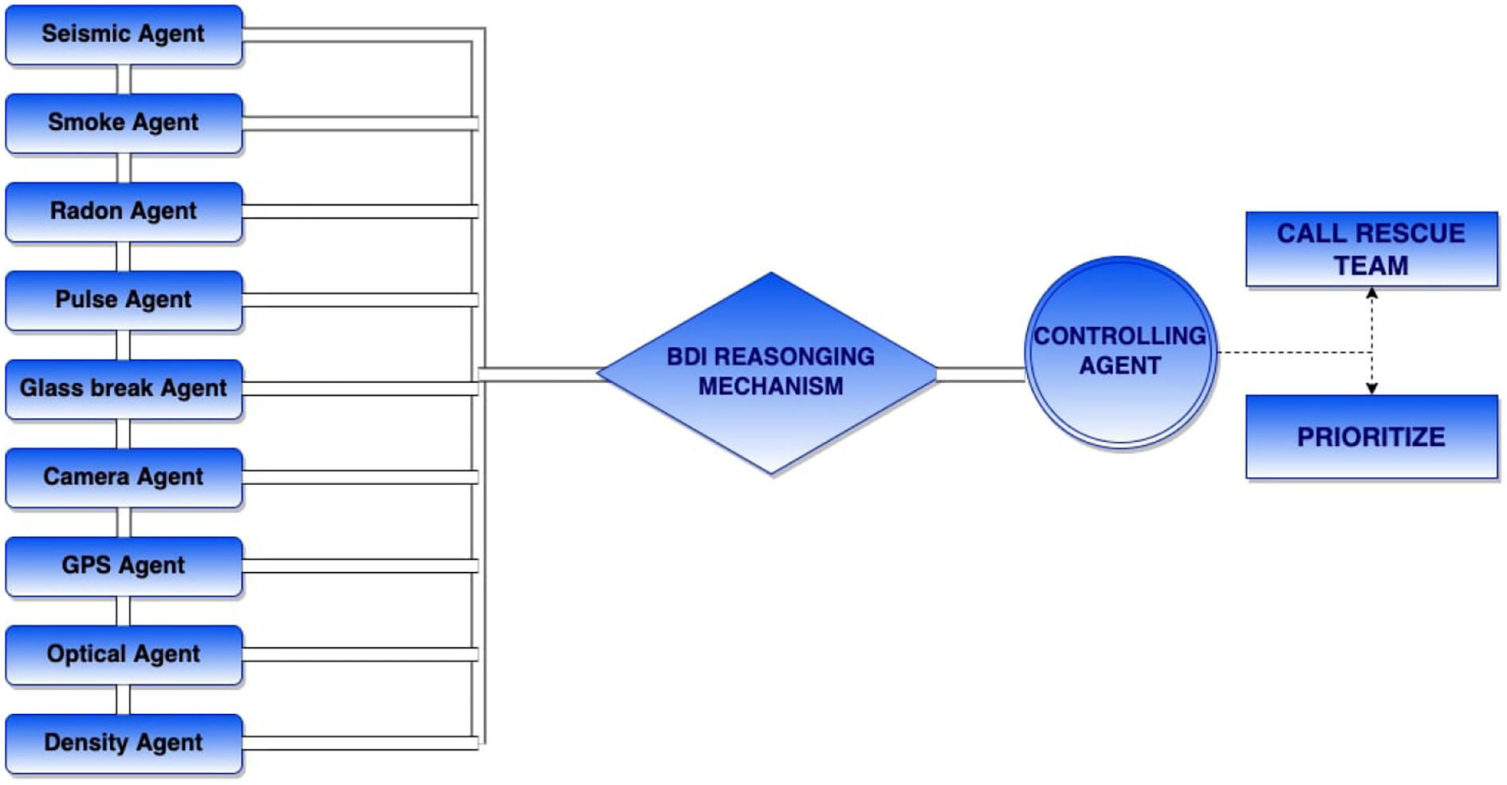
Multi agent system (MAS) with business, desire, and intention (BDI) and situation awareness.

As shown in Figure 2, we present a system that has a possible reasoning mechanism with a situation-aware technique to monitor hazardous situations, take decisions intelligently, where agents can collaborate and come up with a precautionary plan within time to control the disaster situation. The primary roles of agents are to precept the surroundings and perform the reasoning mechanism to model the decision support system with the situation-awareness technique.

The system is based on a BDI agent where an agent can plan the next step according to their belief set. We introduce the situation-awareness concept to the BDI agents to enhance their capability to identify the essence of the problem in the dynamic environment according to their belief set, come up with the following action and trigger the plan.

We assume that every sensor collaborates with its correlative BDI agent, where the agents acquire new knowledge from the agents/sensors or have beliefs based on their existing knowledge. For instance, whenever an agent acquires the data from the correlative sensor, it checks the information based on the belief set and plans accordingly. The belief set of agents can change dynamically concerning the facts agents hold about the world.

Moreover, every agent in the system is specifically designed and assigned tasks to achieve the goals. BDI mechanism is the goal-directed approach where the belief set of agents can be revamped whenever the situation changes. However, due to the dynamic environment, the most recent value of the belief is stored in the agent's memory whenever the belief set updates. Intrinsically, actions are the agent's intentions that the agent has to execute to manage the disaster environment. Every intention originated in its stack of actions. After the perception of the situation, the plan starts to execute whenever the event occurs. Every agent generates the optimal plan to acquire the desirable goals. New intentions can be formed due to the external event occurrence in the system. For edge communication modeling, agents can communicate with each other only to exchange information or keep each other updated about the situation, but it also depends on their intentions to communicate.

When each agent is aware of the situation and performs its reasoning mechanism to come up with the optimum plan, in the end, human assistance is required to save the victims.

For instance, if the fire detection is acquired by agent ^*i*^, it matches the acquired context with its belief set and starts executing the actions concerning agent ^*i*^'s intentions. According to this concept, agent ^*i*^ has the intention to inform the corresponding agent ^*j*^ about the fire detection in order to keep agent ^*j*^ updated and give the alert to the agent; thus, the agent can perform its assigned tasks, e.g., check the human presence in the fire detected building. Based on this example, every agent knows the environment, comprehends the information, and develops an optimistic plan. Finally, rescue teams are required to help the people.

Therefore, the system consists of two modes, namely the automation mode and semi-automation mode. In automation mode, the agents perform their reasoning according to the facts they gather from the environment and plan to act efficiently. In the semi-automation mode, agents collaborate with the rescue teams according to their priority level; thus, the rescue team can take the lead.

Moreover, the following agents are used in our proposed model concerning our case study:

- Seismic Agent: For earthquake detection, it acquires information from the seismic sensor.
- Density Agent: After the earthquake detection, the seismic agent alerts the density agent to ensure the human is present in the earthquake-detected area.
- Glass Break Agent: Seismic agent alerts the glass break agent for minor damage detection.
- Pulse Agent: If minor damages occur, the glass break agent invokes the pulsing agent for significant damage detection that can cause the building to collapse.
- Smoke Detector Agent: It gets the data related to fire if the earthquake damage has caused any. If so, it sends an alert to the density agent for human count detection.
- Radon Agent: Likewise, the radon agent collects information on gas leaks if the earthquake damage has caused any. If so, it calls the radon agent to collect the data on the human count in that area.
- Camera Agent: The camera agent uses live streaming in the area where the earthquake hits; therefore, security teams can help the victims before the rescue team arrives.
- GPS Agent: GPS agent is used to get the location where the disaster occurs.
- Optical Agent: The agent calls the density agent if any rainfall activity overflows the water in a dam to check the human presence in the area.
- Controller Agent: The core of the system checks the severity level of the data and prioritizes which rescue teams should be called.

After acquiring the situation awareness, each agent performs their reasoning mechanism and takes the desirable plan to achieve it.

## 4. Controller agent reasoning

The proposed algorithm consists of BDI-Agents that depend on their in-built reasoning capabilities to gather data, process it, and act accordingly. Each BDI-Agent is provided with various values, namely, severe, medium, and mild, in their belief sets. In the intention part of the BDI-Agents, the agents alert the controller agent regarding the extent of the calamity. If the current value falls within the severe range, the situation is deemed severely catastrophic. If the current value falls within the medium value range, then the situation is deemed catastrophic but not too much. The situation is deemed mildly dangerous if the current value falls within the mild value range. In all these three scenarios, the controller agent is responsible for contacting the relevant authorities. The controller agent is a BDI-Agent with the list of relevant authorities in its belief set and the method of contacting them in its intention set. When the controller agent receives an alert from any other agent, it sends the relevant authorities accordingly.

Once the system has alerted the relevant authorities regarding the calamity and its severity, it is up to the authorities to decide whether to take action. This decision is communicated with the system using flag values. If the authorities decide to take any action, the flag value becomes 1, and the system halts, i.e., the agents stop being autonomous since the authorities will now control them accordingly. However, if the authorities decide not to take action, the flag value becomes 0, and the system continues with all the agents working as before their corresponding tasks.

**Algorithm 1:**
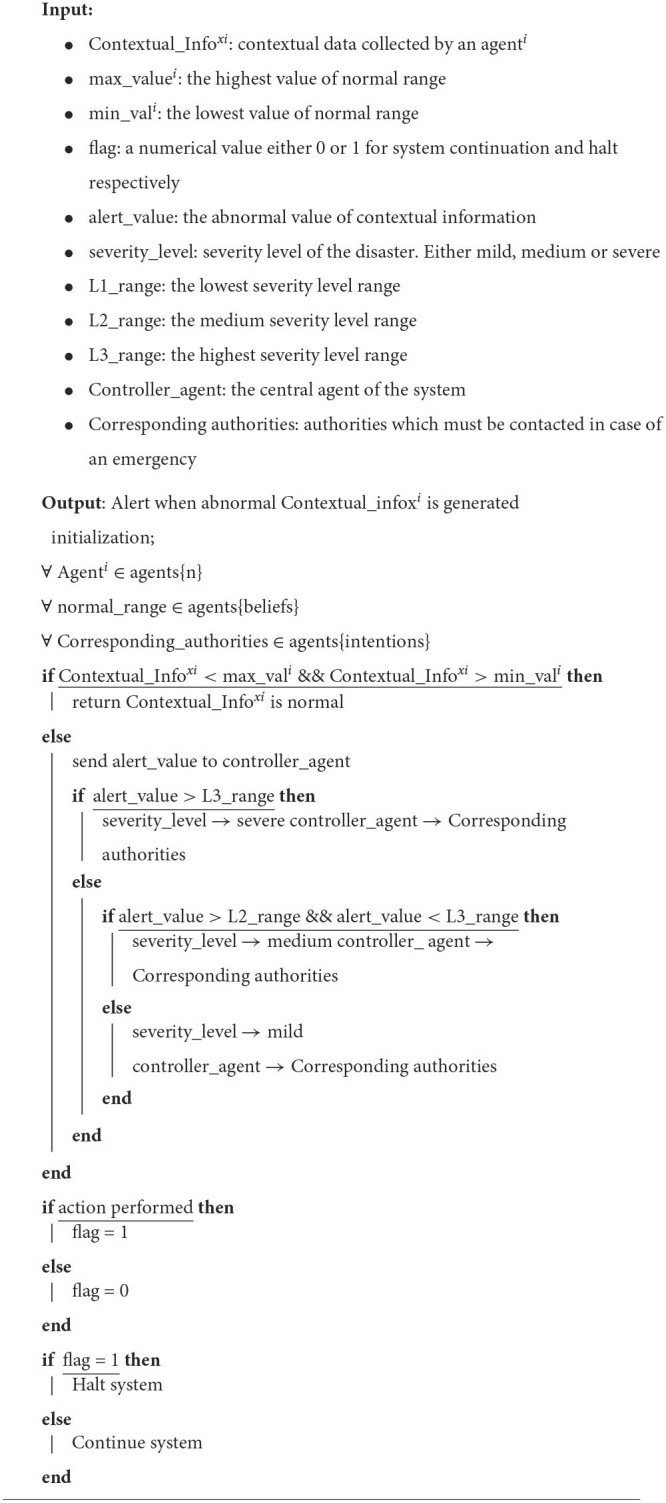
Reasoning algorithm.

## 5. Context modeling of MAS using ontology

**Figure 3:**
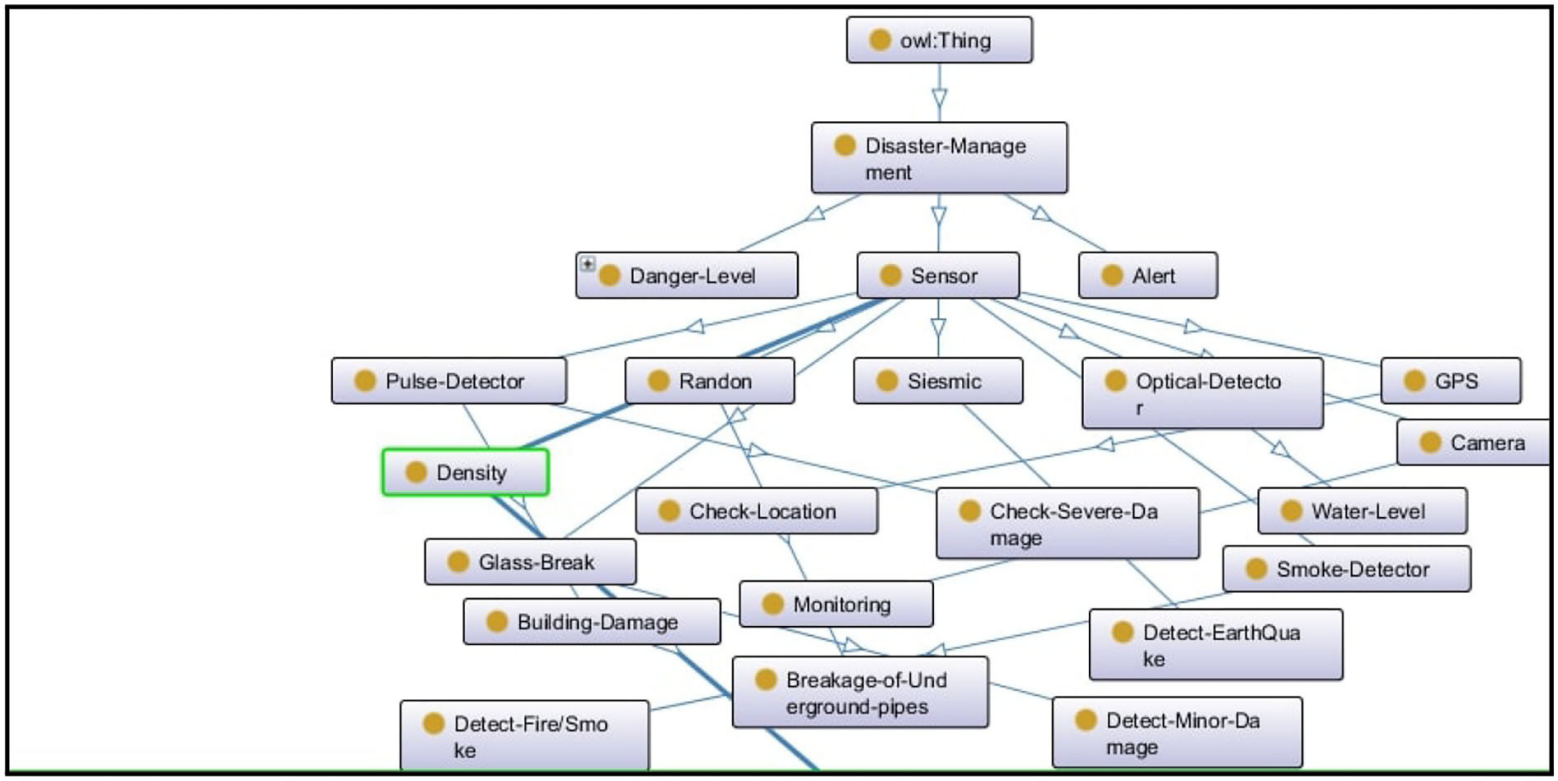
Onto-graph to show the system map.

**Figure 4:**
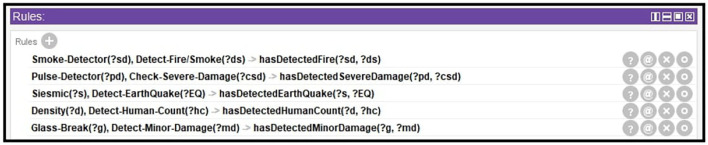
Predefined set of rules for agents.

Utilizing the smart environment applications trend has increased due to the recent development of the context-aware intelligent system. Significant effort has been made to represent the formal structure of a context-aware system by using the ontology approach (Goel et al., 2017; Barzegar et al., 2019). In the proposed system, we have developed an ontology (OWL 2) in which that sensor's data goes into ontology where intelligent agents [a1, a2 … a_*n*_] can access the data and perform reasoning on it to infer the desired result. Figure 3 shows the structure of the proposed system. Moreover, Figure 4 shows the predefined rules for agents in their internal memory or knowledge in which they decide whether the condition/belief is true or not for further actions.

**Figure 5:**
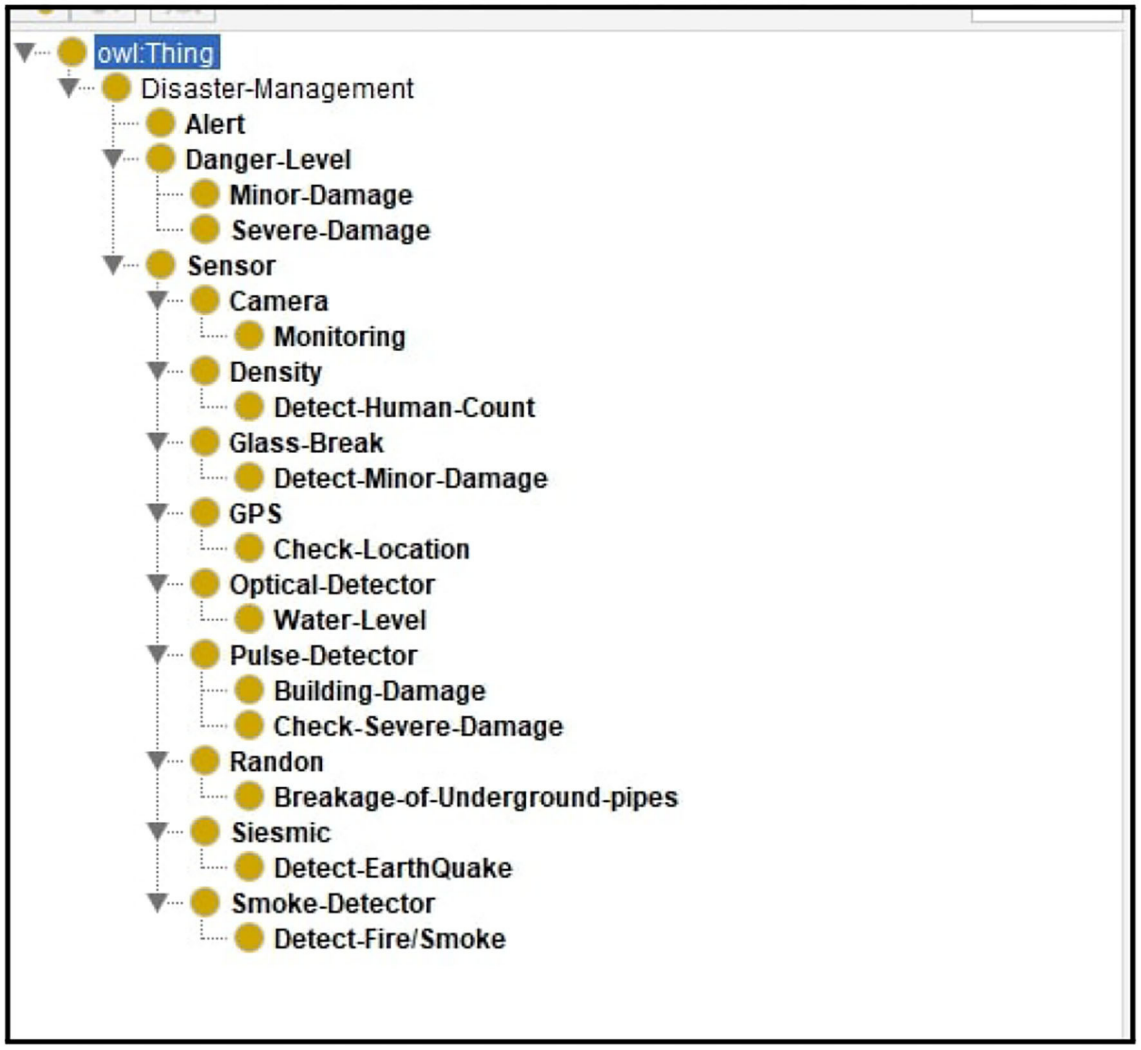
Class-Hierarchy of disaster management system.

Similarly, Figure 5 shows the class hierarchy of the disaster management system according to the sensors/agents and their required actions.

## 6. Prototypal implementation

This section covers the prototypal implementation of the proposed work. The prototype is developed to check the validity and correctness of this work. The physical implementation of this work is currently not possible due to time and technical constraints. For the prototypal implementation of this work, we have opted for the ThingSpeak platform. ThingSpeak is an IoT analytics platform service that allows the aggregation, visualization, and analysis of the live data streams by agents connected to it. These agents can be physical or virtual. Using ThingSpeak, multiple other platforms such as Twitter, IFTTT, Twilio, MATLAB, etc., can be easily integrated into a system. ThingSpeak acts as a bridge between other platforms and the system. To send data to the system, we developed several virtual intelligent agents. The codes of these agents were written in the Python programming language, a general-purpose programming language that is capable of developing complex systems. Not only this, but nowadays, python is proving to be a strong programming language in domains such as image processing, AIoT, etc. This is due to its ability to simplify complex tasks using indentations, thus improving code readability and allowing a better understanding of the developed system.

For prototypal implementation, seismic, density, and fire agents were developed in a python programming language using Notepad++ editor (Gladkauskas, 2019). The agent was connected with the ThingSpeak website channel, which allows the visualization of data sent by the agent. This data is saved on the ThingSpeak cloud (Mohamad et al., 2019). ThingSpeak is integrated with MATLAB, thus allowing for the analysis of data received on the ThingSpeak website. MATLAB has also been used to generate an alert email and send it to the user's mail account (Muqeet, 2019). Other than this, an alert notification is sent to the user's chosen device with the help of the IFTTT application. The IFTTT application is installed on the chosen device, and a notification can be triggered on the IFTTT website and sent to the IFTTT application on the device to generate a notification when a specific situation occurs during data acquisition (Miry and Aramice, 2020). Furthermore, a message is sent using the Twilio application. This application sends a message on a specified number when a specific condition is met during data acquisition.

Even though physically implementing the proposed framework is not possible currently, several pieces of literature have been reviewed to find out how this framework can be implemented if ever need be. This explanation provides a clear picture to the reader of how data can be acquired and processed in this framework when using physical sensors (Abdulwahid, 2019; Tapakire and Patil, 2019).

### 6.1. ThingSpeak channels

**Figure 6:**
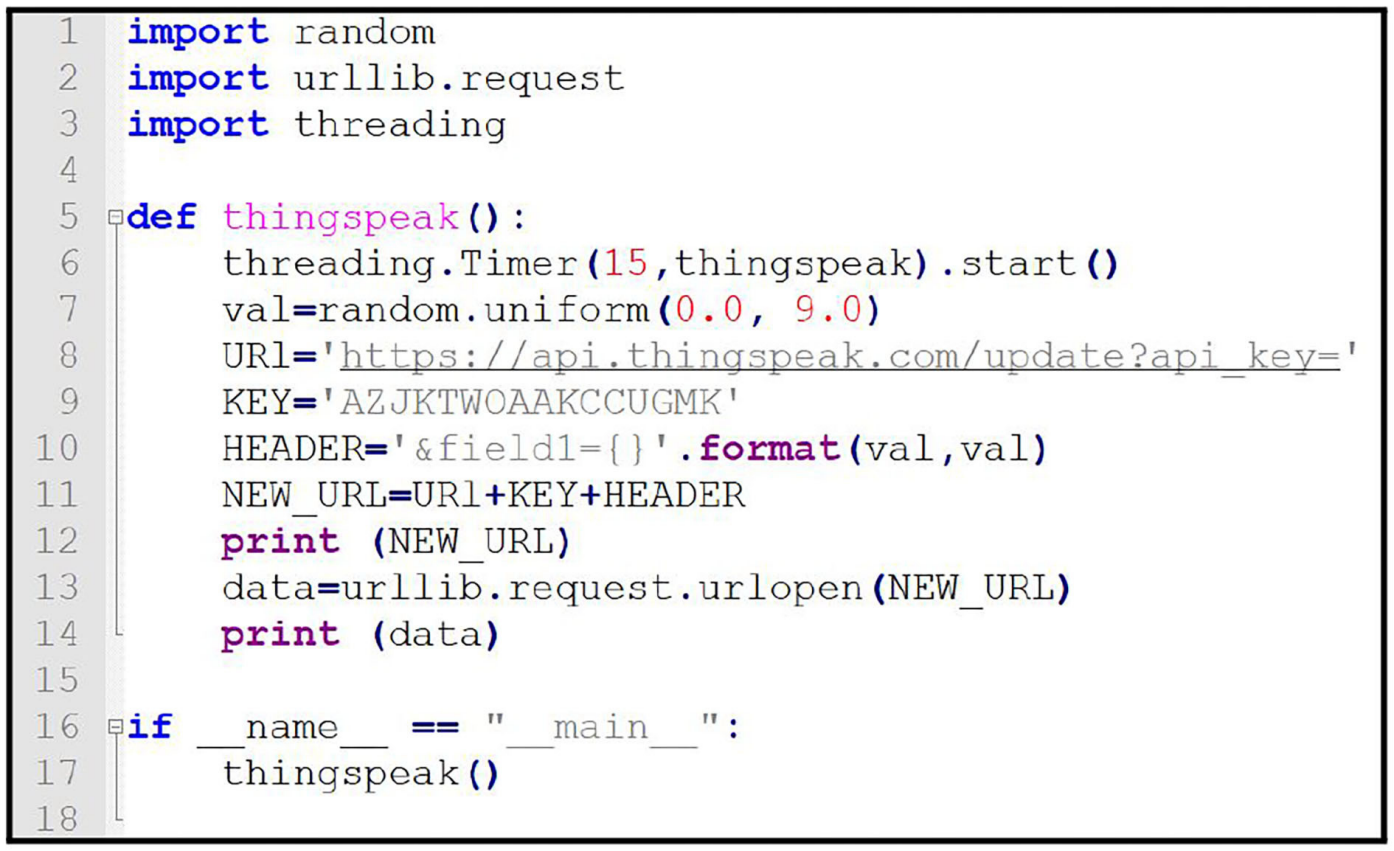
Python code for the seismic agent responsible for monitoring earthquakes.

For the prototypal implementation of the proposed work, a ThingSpeak channel named “Disaster Management System (Control Agent)” was made. A seismic agent gathering data about the magnitude of earthquakes has been developed in a python programming language using notepad++ editor for this work. Figure 6 shows the code of an agent by which an agent is responsible for making decisions.

**Figure 7:**
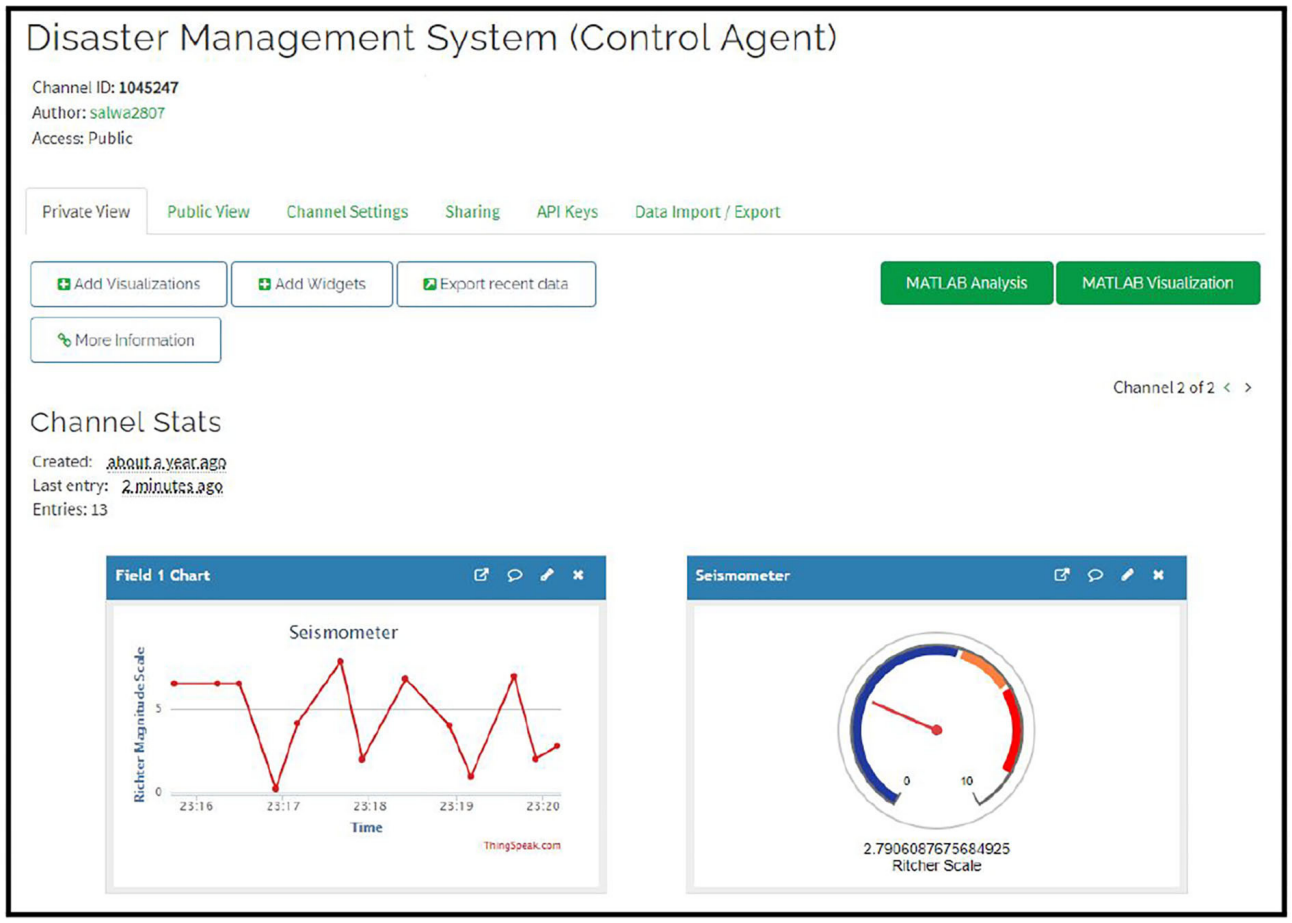
The ThingSpeak channel created for receiving data from multiple different agents of the system. This channel can also be called control agent as it receives and manages the information in the system.

Figure 7 shows the channel developed for this work. The figure shows the name of the channel developed, along with the unique channel ID. Every channel in ThingSpeak is provided with its unique channel ID, which can be used for accessing the channel during sending and retrieving of data. The channel also contains information about when the channel was created, when the last entry was made, and the total number of performed entries. Access to these channels is public, so anyone with the channel ID or the link can easily access it. Other than this, the access can be made private so that only the user can access it, or access to the channel can be given to specific people by sending the channel information to their email address.

**Figure 8:**
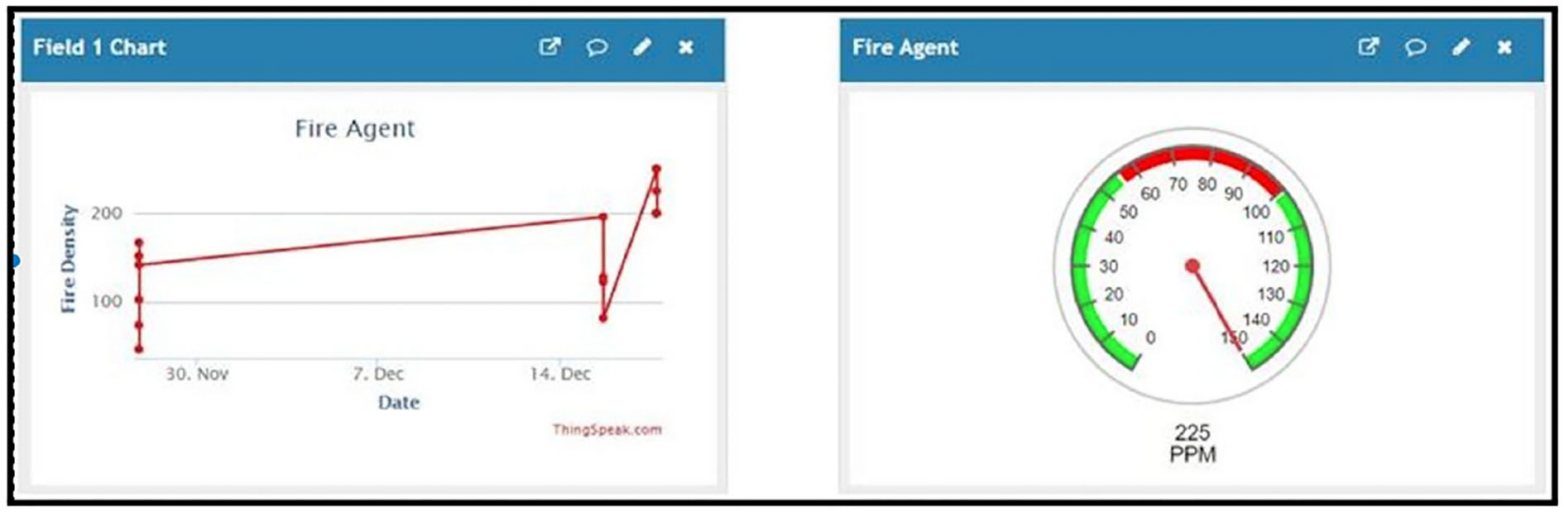
The data sent by some of the different agents developed for the proposed system.

Figure 8 shows the agents on the ThingSpeak channel. The agents send their collected data to the ThingSpeak channel. The ThingSpeak channel fields receive this data and display the current value.

### 6.2. ThingSpeak react

ThingSpeak react is an application associated with ThingSpeak. It generates a “reaction” using the data from the ThingSpeak channel. This reaction can be an email, a notification on a device, or a tweet. For this work, an SMS, an email, and a notification have been used.

#### 6.2.1. EMAIL

**Figure 9:**
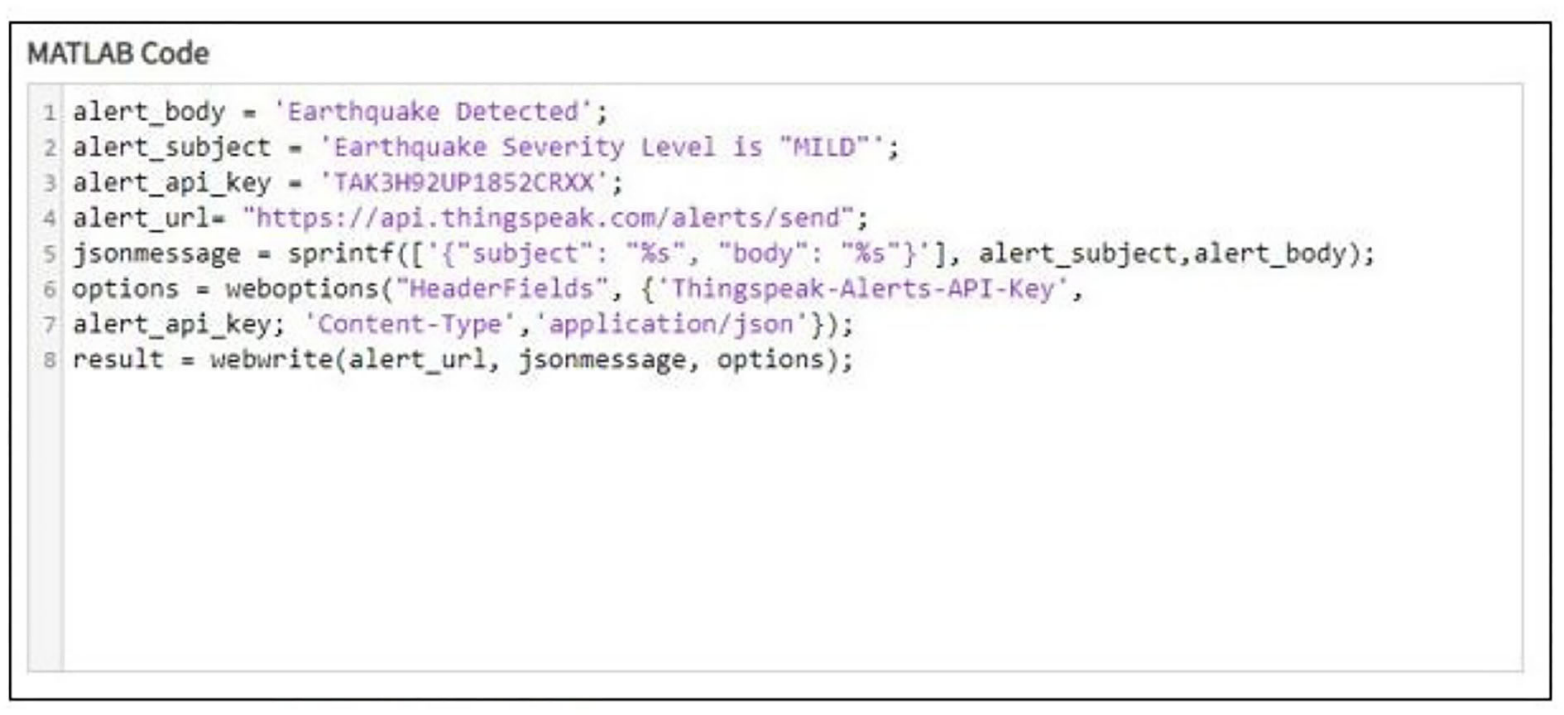
MATLAB code written for sending an email alert to the corresponding person.

When the received data is above or below a certain point, an email is generated and sent to the email address associated with the owner of the ThingSpeak account. This task is achieved using the MATLAB code shown in Figure 9.

**Figure 10:**
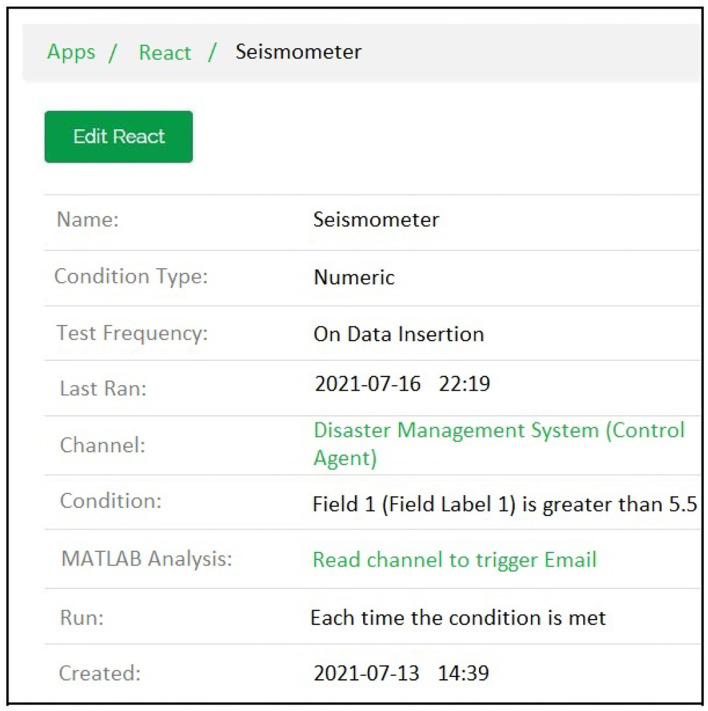
Email generation react created for connecting the MATLAB code with the ThingSpeak channel.

**Figure 11:**
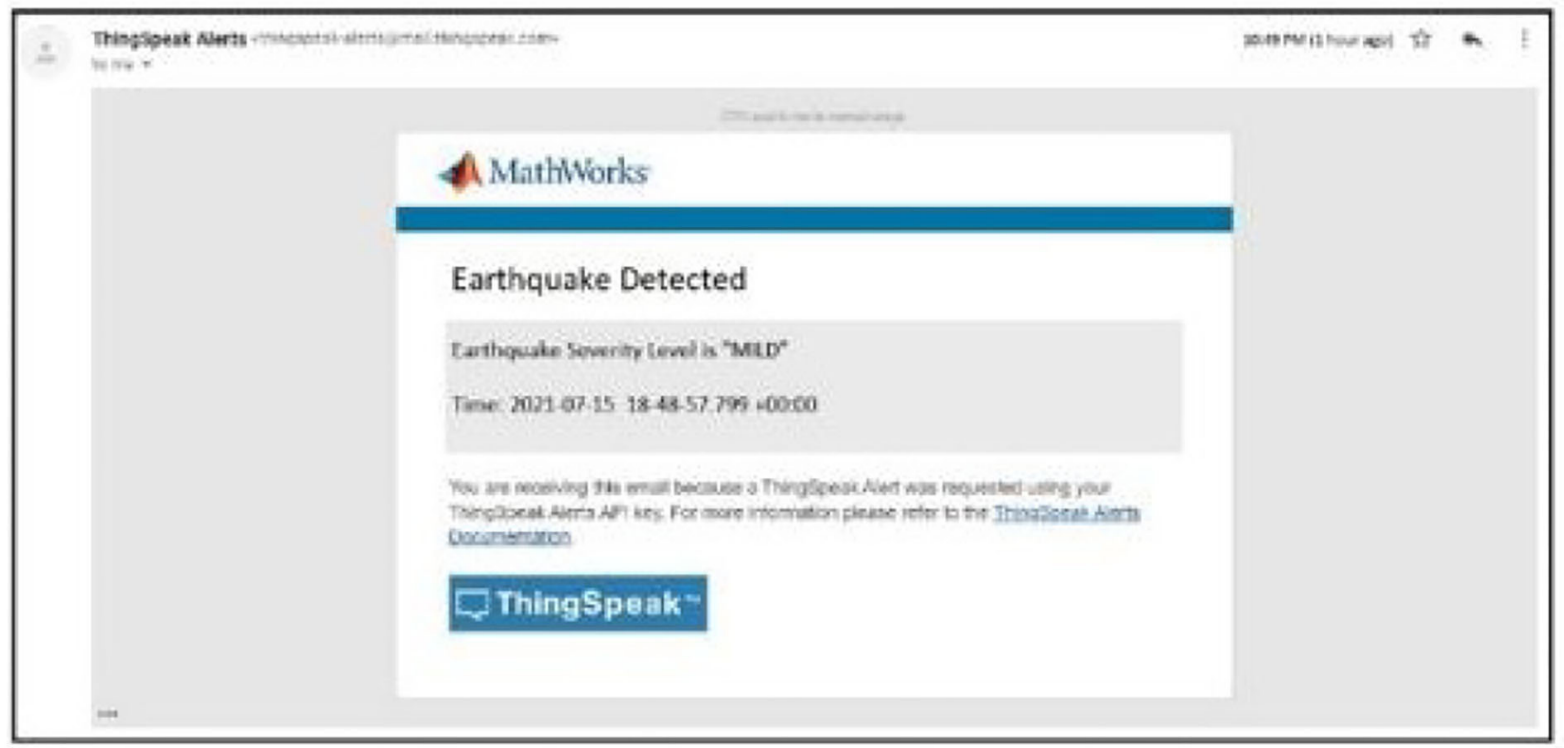
The Emails Received when pre-defined agent data value conditions were met.

**Figure 12:**
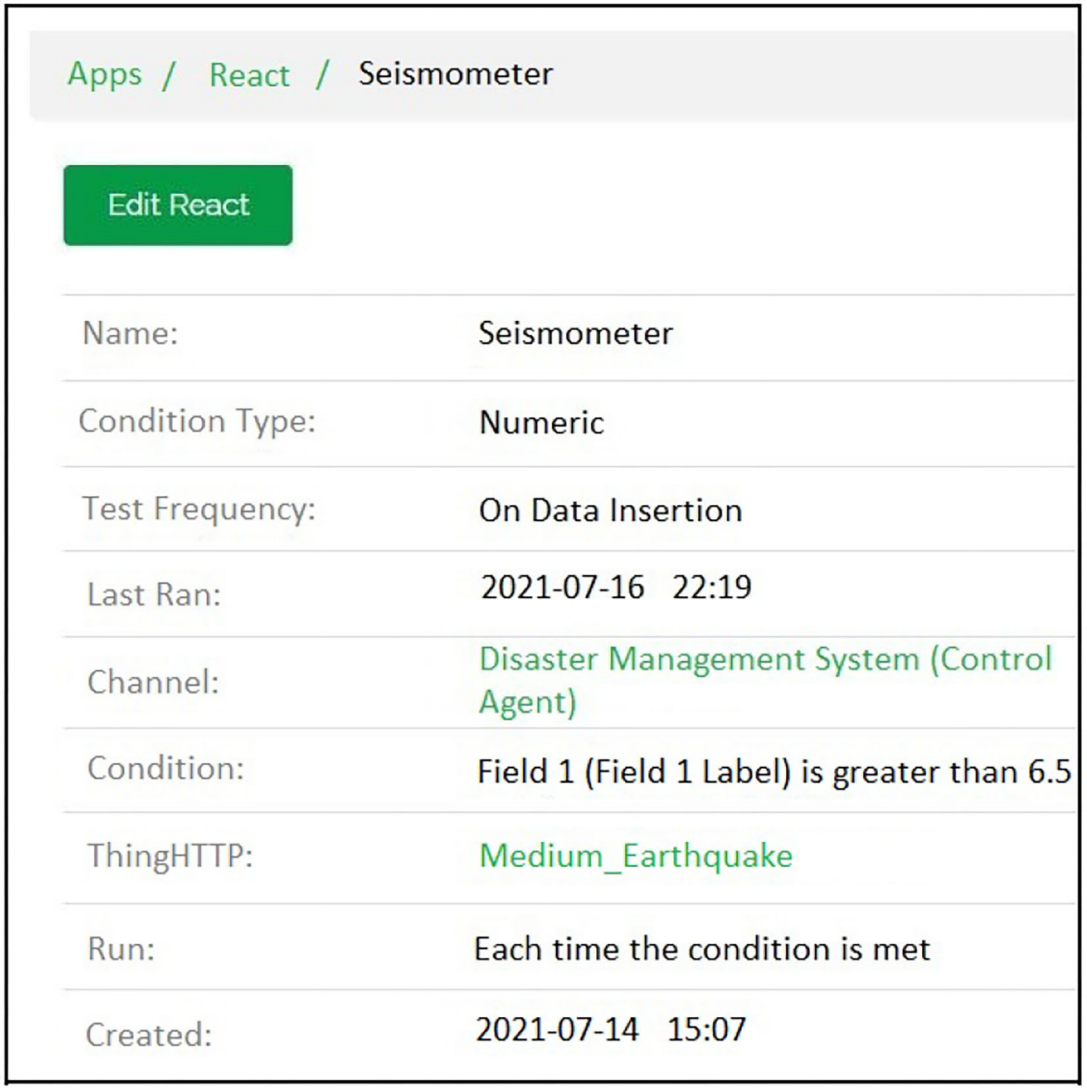
IFTTT notification react created for connecting ifttt platform with the thingspeak channel.

The email generation react creation regarding the detection of earthquake can be seen in Figure 10 while Figure 11 shows the resultant email received about the earthquake detection and Figure 12 shows the notification created while detection.

#### 6.2.2. Notification

**Figure 13:**
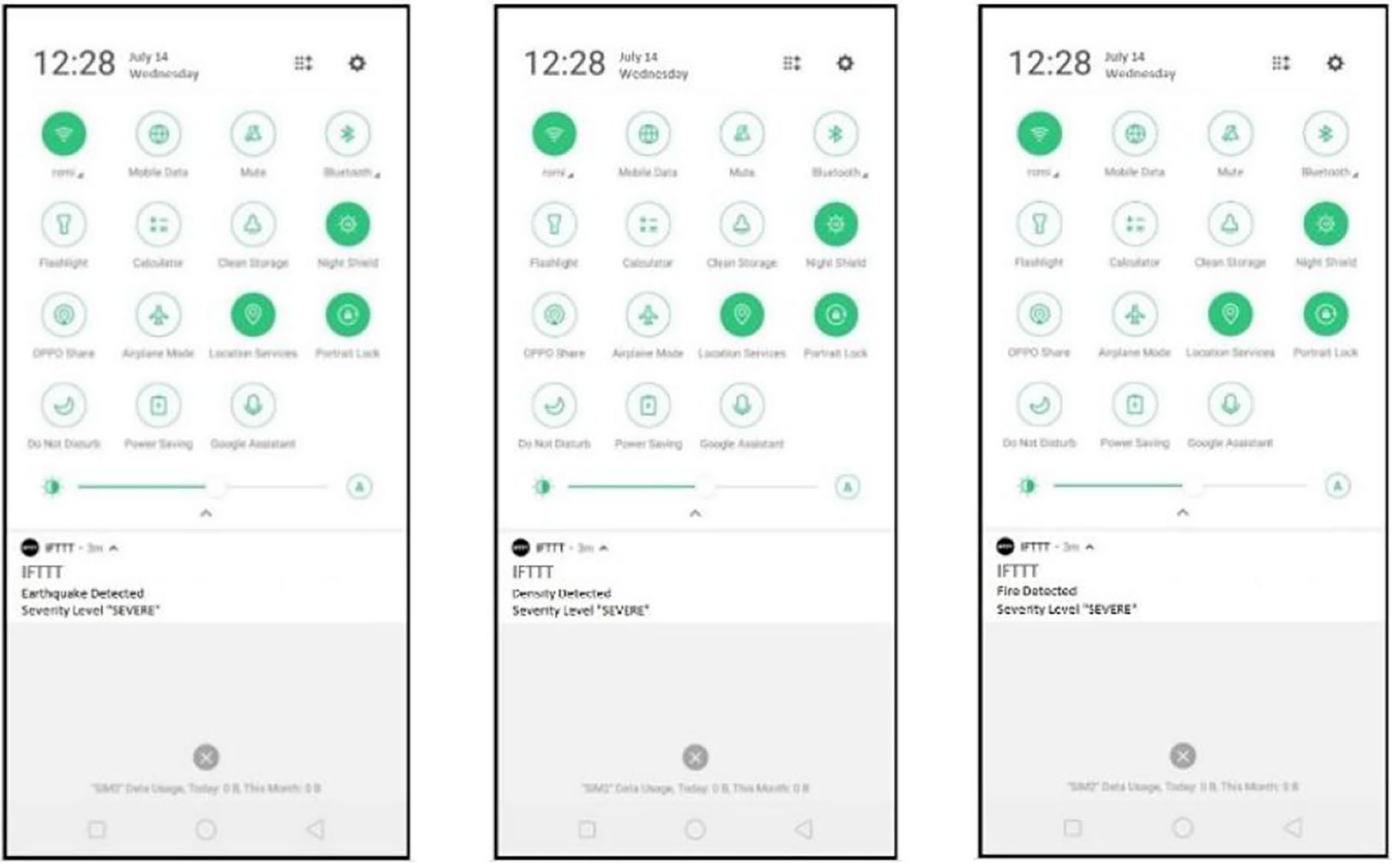
IFTTT notifications received in the mobile phone's notification panel.

**Figure 14:**
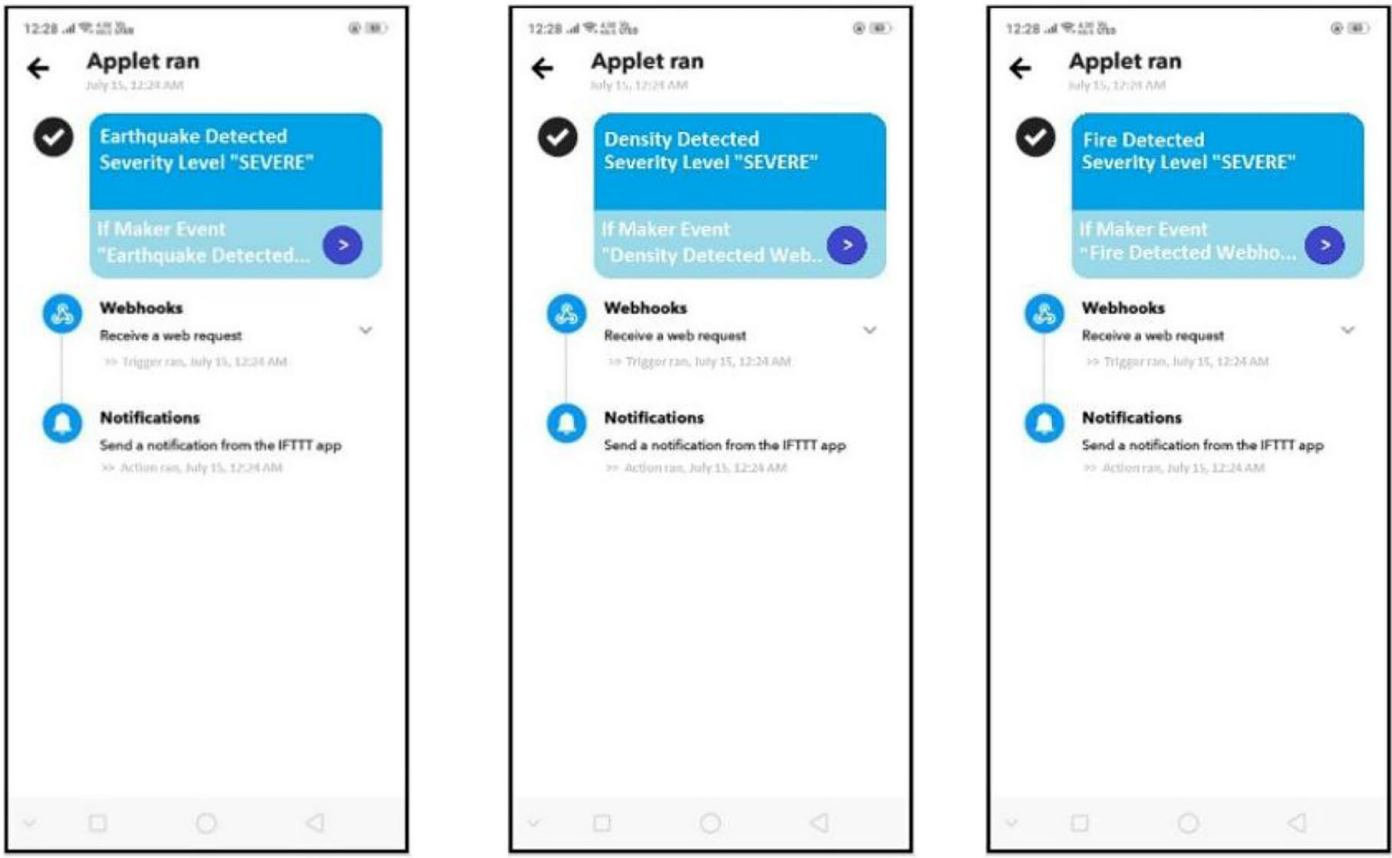
IFTTT notification in the IFTTT application.

In addition, a notification is sent to the device of the account owner (Rescue team). This is done using the IFTTT applet. The device's connection is established by installing the IFTTT application where the notification is received. Regarding the earthquake severity, the notification generation react creation can be seen in Figure 13. In contrast, Figure 14 shows the resultant notification received in the notification panel of the disaster scenario. The IFTTT application is installed on the mobile device, respectively.

## 7. Conclusion

This work proposes a framework that can handle the hazardous situation caused by natural or man-made disaster. This framework is based on the BDI reasoning mechanism and situation-awareness technique to provide an efficient energy consumption, memory limitation, and optimal planning system. Using these two together leads to better decision-making and allows the system to adapt its behavior according to the environment. We have utilized an ontology-driven approach for structuring the system, thus providing a clear understanding of the flow of information within the system. Also, we have proposed a prototypal implementation of the system using ThingSpeak, MATLAB, Twilio, and IFTTT platforms, thus proving the correctness of the system (i.e., proving that this system can be physically implemented in a real-world scenario).

## Data availability statement

The original contributions presented in the study are included in the article/supplementary material, further inquiries can be directed to the corresponding authors.

## Author contributions

KS and SA: conceptualization. SA: data curation, investigation, methodology, and software. YZ and SA: formal analysis. SK and RA: funding acquisition. AA, YZ, and RA: project administration. SA and MN: supervision. AA, SA, MN, YZ, RA, and SK: validation. YZ: resources, visualization and writing-review and editing. All authors contributed to the article and approved the submitted version.

## Funding

This research was supported in part by Basic Science Research Program through the National Research Foundation of Korea (NRF), funded by the Ministry of Education (NRF-2021R1A6A1A03039493), and in part by the NRF grant funded by the Korean government (MSIT) (NRF-2022R1A2C1004401).

## Conflict of interest

The authors declare that the research was conducted in the absence of any commercial or financial relationships that could be construed as a potential conflict of interest.

## Publisher's note

All claims expressed in this article are solely those of the authors and do not necessarily represent those of their affiliated organizations, or those of the publisher, the editors and the reviewers. Any product that may be evaluated in this article, or claim that may be made by its manufacturer, is not guaranteed or endorsed by the publisher.
